# New Compact 3-Dimensional Shape Descriptor for a Depth Camera in Indoor Environments

**DOI:** 10.3390/s17040876

**Published:** 2017-04-16

**Authors:** Hyukdoo Choi, Euntai Kim

**Affiliations:** 1 School of Electrical & Electronic Engineering, Yonsei University, Seoul 03722, Korea; goodgodgd@yonsei.ac.kr; 2 LG Electronics, Seoul 08592, Korea

**Keywords:** shape descriptor, correspondence, point cloud, principal curvature

## Abstract

This study questions why existing local shape descriptors have high dimensionalities (up to hundreds) despite simplicity of local shapes. We derived an answer from a historical context and provided an alternative solution by proposing a new compact descriptor. Although existing descriptors can express complicated shapes and depth sensors have been improved, complex shapes are rarely observed in an ordinary environment and a depth sensor only captures a single side of a surface with noise. Therefore, we designed a new descriptor based on principal curvatures, which is compact but practically useful. For verification, the CoRBS dataset, the RGB-D Scenes dataset and the RGB-D Object dataset were used to compare the proposed descriptor with existing descriptors in terms of shape, instance, and category recognition rate. The proposed descriptor showed a comparable performance with existing descriptors despite its low dimensionality of 4.

## 1. Introduction

RGB-D sensors with affordable prices and decent performance have been available since 2010, and a new era in 3D computer vision and robotics has begun. There has been tremendous progress in research dealing with 3D data such as human pose and gesture recognition [1,2], point cloud registration [3,4], simultaneous localization and mapping (SLAM) [5], and object recognition [6]. In these studies, a vector that encodes distinctive property of local region, called a descriptor, plays an important role where descriptors are usually used to find correspondences [3,4,7,8] between two images or to encode a higher level descriptor for objects or scenes.

For 2D images, a number of descriptors have been proposed. After the monumental work of SIFT [9] and SURF [10], many researchers have competed for the best descriptor in terms of distinctiveness, processing time, and robustness to changes in transformation, noise, and illumination. BRISK, BRIEF, and FREAK [11,12,13] are currently popular because of their lower computational burden and better matching performance.

For 3D point cloud, shape descriptors adopted the legacy of 2D descriptors to encode the local shape of a point cloud. Popular options are Spin Image [14], fast point feature histogram (FPFH) [15], signature of histograms of orientations (SHOT) [16], and Tri-Spin-Image (TriSI) [17]. We tested these descriptors, but found they were not as discriminative as the 2D descriptors despite their lengthy descriptor sizes. Another important observation was that the local shapes from RGB-D sensors had limited complexity compared to 2D image patches. We concluded that existing shape descriptors have many redundant dimensions, and this motivated us to design a new descriptor that was as short as possible but as effective as existing descriptors.

The purpose of this study is three-fold: (1) to determine why existing shape descriptors become redundantly lengthy; (2) to analyze the cause of and to quantify the redundancy of high-dimensional descriptors; and (3) to propose a new efficient descriptor and prove the effectiveness of the new descriptor through experimentation. The proposed descriptor is derived from principal curvatures and concatenated with the gradients of curvatures. We found that this 4D descriptor was as effective as those mentioned above.

The paper is organized as follows: Section 2 reviews the existing shape descriptors in a historical context to determine the origin of the redundancy and proposes a new approach for local shape descriptors. A descriptor reflecting the new approach is presented in Section 3 and is compared with existing descriptors in terms of discriminative power in Section 4, followed by conclusions.

## 2. Derivation of a New Shape Descriptor

To understand the popularity of lengthy shape descriptors, we reviewed the historical progress from 2D image descriptors to 3D shape descriptors. Then we statistically evaluated the redundancy of the shape descriptors, and presented here a new approach for local shape descriptors.

### 2.1. Legacy of 2D Image Descriptors

Most of the image descriptors mentioned in Section 1 typically have dimensionality of 128 or 256. A dimensionality of $2^{n}$ is preferred for processing and memory efficiency. Previous studies have proven that correspondence matching accuracy tends to increase with dimensionality, but saturates around 128 or 256 [10,12]. From these results, we think a ‘myth’ was created that greater dimensionality resulted in better performance. However, as we proved in Section 2.3, this is not true for shape descriptors.

### 2.2. Brief Review of Shape Descriptors

Although many local shape descriptors have been proposed, we review here only a selection of notable works comparable to our descriptor. A thorough review of local shape descriptors was given in [18].

Spin Image [14] is one of the most famous shape descriptors. It encodes neighboring points as a radial distance from a normal line through a keypoint and a height from the tangent plane in a cylindrical local frame. Spin Image was built by constructing a 2D histogram of the distances and heights of the neighboring points. Several methods to improve the discriminative power of Spin Image have been proposed [17,19,20]. Pasqualotto et al. [20] proposed to combine color and shape Spin Images to compare two 3D models where similarities of two types of Spin Images are aggregated by fuzzy logic. TriSI [17] is one of the latest variations. To estimate TriSI, three spin images are computed along the three axes of the local reference frame (LRF) and concatenated into a single vector, and then the dimensionality of the vector is reduced by the principal component analysis (PCA) approach.

The point feature histogram (PFH) [21] is another important descriptor and the basis of the more popular descriptor, FPFH [15]. For every pair of points in the vicinity of a keypoint, a unique LRF is estimated, and then the three angular signatures of the pair are computed. The three histograms are made from the three signatures independently and concatenated to output the PFH descriptor vector. Since the PFH is computationally expensive, FPFH was proposed to reduce complexity and is now one of the most popular descriptors. The differential FPFH (dFPFH) descriptor [22] is the latest variation that captures surface irregularities by concatenating a difference vector between the FPFHs of inner and outer spheres.

The SHOT descriptor [16] partitions the local spherical space by the azimuth, elevation, and radial distance in the keypoint’s LRF. A histogram of angles between the neighbor’s normal vectors and the z-axis of the LRF is constructed for each space bin, and then the histograms are quadralinearly interpolated and concatenated to complete the SHOT descriptor. It outperformed Spin Image and FPFH in keypoint matching, but it is vulnerable to variations of resolution. SHOT is readily expandable to RGB-D data by adopting a texture-based histogram called color SHOT (CSHOT) [23].

Although the popular shape descriptors have colored versions, we do not discuss them. Color information is easily adopted to shape descriptors simply by concatenating shape and texture descriptors.

Other notable individual works include 3D shape context [24], intrinsic shape signature [25], rotational projection statistics [26], normal aligned radial feature [27], and binary robust appearance and normal descriptor (BRAND) [28]. Among them, BRAND has a similar motivation to ours and pursues a robust, fast, and memory efficient descriptor applicable to implementation in mobile phones and embedded systems. The descriptor consists of 256 binary relationships between 256 pairs of points in the vicinity of a keypoint. The dimensionality is high, but it takes only 32 bytes. Despite its memory efficiency, it outperformed CSHOT and Spin Image in keypoint matching.

Recently, data-driven approaches such as the convolutional neural network (CNN) have become popular to automatically extract discriminative features. The CNN approaches have successfully detected and classified objects [29,30,31] from images where the output of CNN usually worked as a global descriptors of objects. Some works [32,33] tried to use CNN for local patch retrieval. Outputs of middle layers of a network trained for object classification were used as patch descriptors. However, to our best knowledge, CNN features as a local descriptor have been tried only for RGB images but not for local shapes. Therefore, CNN features are still in different domain from local shape descriptors and more suitable for global descriptors. Though there are a number of global descriptors [34,35,36] to query the entire object, they are out of the scope of this study. Local descriptors have a unique role, for instance, point-to-point matching in point cloud registration.

### 2.3. Redundancy of Shape Descriptors

To analyze the amount of redundancy of shape descriptors, we estimated the number of effective principal components (nEPC). The nEPC counts the number of eigenvalues of a descriptor covariance matrix which are larger than $\lambda_{1}*0.01$, where $\lambda_{1}$ is the largest eigenvalue. The nEPC roughly estimates how many dimensions were actually used. Three RGB-D image sequences in the CoRBS dataset were used to estimate the nEPC: Cabinet, Desk, and Human [37]. The description of the dataset is given in Section 4.1. In each sequence, the five types of descriptors, FPFH, SHOT, Spin Image, TriSI and BRAND were extracted from 10,000 randomly sampled points, and the eigenvalues were computed from the covariance matrix of descriptors for each descriptor type in each sequence. Table 1 shows the results, where rEPC stands for the ratio of effective principal components.

Two observations were made. For the first four descriptors from FPFH to TriSI, rEPCs were generally low from 5.2% to 27.3% which indicates that the descriptors occupy excessive memory with no effect. Their nEPCs grew with increment of dimensionalities, but the rEPCs decreased. Consequently the information in each dimension shrank as descriptor length increased. Second, BRAND had exceptionally high rEPC despite its high dimensionality because a BRAND descriptor is comprised of 256 independent binary tests while the others have highly correlated dimensions. However it still wasted more than half of total dimensions. Therefore, higher dimensionality provided limited benefit considering its excessive memory burden.

### 2.4. New Approach for Shape Descriptors

Here we propose a new approach for local shape descriptors specialized in recognizing shapes captured by affordable depth sensors in an ordinary environment. First, shape descriptors do not have to express highly complicated shapes. Object surfaces are not so complex in ordinary environments such as houses, offices and stores. Most of them are almost flat or simply curved. In addition, although depth resolution of depth cameras has been improved, there is still noise more than a few millimeters depending on situation [38] and a depth camera only captures a single side of a scene. Complex objects usually have small and peaky parts but they look relatively simple in a depth image. That is the reason that many previous works [14,16,17,22,26] used finely reconstructed 3D models from publically available datasets.

Second, since keypoint matching is not useful for shapes, short descriptors are preferred. In RGB images, where pixel values are highly dynamic, key points are readily detected and tracked. On the contrary, depths usually change smoothly over a continuous surface, so detecting and tracking key points is relatively difficult. Local shapes are rarely unique and peaky shapes easily look different from a different view pose. Accordingly, dense matching is more effective than key point matching thus descriptors have to be computed at almost all points. To compare crowded descriptors with limited processing power and memory, like in mobile phones or robots, short descriptors are more advantageous.

Third, local descriptors should be easy to understand, implement and reproduce. Since computing local descriptors is just beginning of an application, it should not burden users. The popular shape descriptors [14,15,16] are easy to understand and require no pre-training or pre-processing more than a smoothing filter and computing normal vectors. On the other hand, for instance, TriSI is not popularly used despite its better performance than Spin Image or SHOT, because it requires a pre-training stage to compress descriptor size by the PCA approach. Pre-training makes the performance of an algorithm to be dependent on training data, which is not always reliable and requires more effort. Following the new approach, we present in Section 3 our new descriptor design based on principal curvatures.

## 3. Principal Curvatures with Gradients

We propose a novel shape descriptor, principal curvatures with gradients (PCWG), which is four dimensional but as effective as existing descriptors. The first two elements are the principal curvatures of the surface, and the second two are the gradients of the principal curvatures along two principal directions. The idea of utilizing principal curvatures for correspondence matching is not completely novel, as proposed in [39] where curvatures are used for outlier rejection in ICP. However, in our method principal curvatures work as a shape descriptor for the first time along with curvature gradients.

Furthermore, we present a novel method to accurately estimate principal curvatures by formulating the estimation problem as quadratic programming (QP). Cheng et al. proposed a principal curvature estimation method based on normal fitting [40]. The curvature values were optimized to fit the normal vectors on the surface rather than the point coordinates, but it required too many intermediate variables. Recently, Spek et al. presented a fast method to estimate curvatures by solving a non-linear optimization problem where the curvature values and normal estimation were iteratively refined [41]. On the contrary, our formulation is derived in an intuitive manner and solved by a closed-form equation.

### 3.1. Curvature Estimation

**Figure 1:**
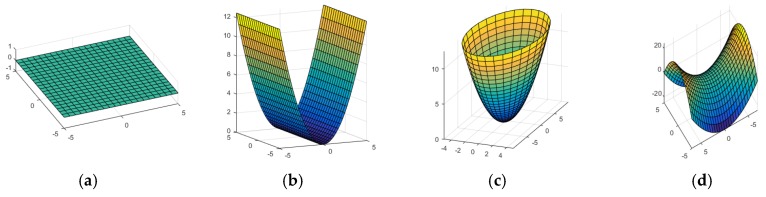
Quadratic surfaces from: (**a**) Plane ($C_{\alpha }=C_{\beta }=0$); (**b**) Parabola ($C_{\alpha }>C_{\beta }=0$); (**c**) Elliptic paraboloid ($C_{\alpha }>C_{\beta }>0$); and (**d**) Hyperbolic paraboloid ($C_{\alpha }<0,\;C_{\beta }>0$).

Provided that an arbitrary point cloud within a certain radius from a specific point is lying on a continuous surface, the intrinsic shape of the point cloud can be modeled by a quadratic surface. The basic form of a quadratic surface is given as follows:

(1) $$z=\frac{1}{2}\left(C_{\alpha }x^{2}+C_{\beta }y^{2}\right)$$

where $C_{\alpha }$ and $C_{\beta }$ are the primary and secondary curvatures of the surface, respectively, and $\left|C_{\alpha }\right|\geq \left|C_{\beta }\right|$. By varying the only two curvatures and applying a rotation, it can express various shapes as depicted in Figure 1. This expression is simple, descriptive, and comprehensive.

Let us derive the curvatures from a given point cloud. When there is a center point $p_{k}$ and a normal vector $n_{k}$ at $p_{k}$, the point cloud around the center point within a radius r is denoted by $\mathbb{P}={\left\{p_{j}\right\}}_{j=1:N}$ where $p_{j}$ is a neighboring point around $p_{k}$. In this subsection, let us assume that the center point is translated to the origin for simplicity, that is, $p_{k}\to 0$ and $p_{j}-p_{k}\to p_{j}$. To generalize (1), it is rewritten in the matrix form and an arbitrary rotation is applied:

(2) $$p^{T}RA_{0}R^{T}p-b_{0}^{T}R^{T}p=0$$

(3) $$p^{T}Ap-b^{T}p=0$$

where $A_{0}=diag\left(\begin{array}{ccc} C_{\alpha } & C_{\beta } & 0 \end{array}\right)$, $b_{0}={\left[\begin{array}{ccc} 0 & 0 & 1 \end{array}\right]}^{T}$, and R⊆SO(3) is a rotation matrix. Since $A=RA_{0}R^{T}$, it can be seen as eigen decomposition of A where the eigen values are $C_{\alpha }$, $C_{\beta }$, and 0 and the corresponding eigen vectors are the columns of $R=\left[\begin{array}{ccc} v_{1} & v_{2} & v_{3} \end{array}\right]\subseteq SO\left(3\right)$. Thus, $b=Rb_{0}$ becomes $b=v_{3}$, which corresponds to the zero eigenvalue. The general equation is constrained by $Ab=Av_{3}=0_{3\times 1}$, $b^{T}b=v_{3}^{T}v_{3}=1$, and $A^{T}=A$. The last constraint is for the orthonormal decomposition ($R^{T}R=I$). Our first goal is to estimate the optimal A and b such that:

(4) $$\begin{array}{l} A,\;b=\underset{A,\;b}{\arg min}\underset{j}{\sum }\left\{p_{j}^{T}Ap_{j}-b^{T}p_{j}\right\}\\ subject\;to\;Ab=0_{3\times 1},\;b^{T}b=1,\;A^{T}=A. \end{array}$$

The curvatures and rotation matrix are computed by eigen decomposition of A. This problem belongs to quadratically constrained quadratic programming (QCQP), but it is not convex because of the non-convex constraints. General QCQP can be relaxed to be semidefinite programming, but it needs a formulation with huge matrices and hence is burdened by a heavy computational load. Since this method opposes our intentions, we performed a trick to ease the problem.

From the geometrical intuition, we replaced b with the normal vector $n_{k}$ since the vector $n_{k}$ corresponds to the z-axis for a quadratic surface. Consequently, Eq. (4) is simplified as:

(5) $$\begin{array}{l} A=\underset{A}{\arg min}\underset{j}{\sum }\left\{p_{j}^{T}Ap_{j}-n_{k}^{T}p_{j}\right\}\\ subject\;to\;An_{k}=0_{3\times 1},\;A^{T}=A \end{array}$$

Now we have a simple QP problem that is convex and has a closed-form solution. To solve the problem in the QP form, A is vectorized as follows:

(6) $$\begin{array}{l} a={\left[\begin{array}{cccccc} a_{1} & a_{2} & a_{3} & a_{4} & a_{5} & a_{6} \end{array}\right]}^{T}\\ where\;A=\left[\begin{array}{ccc} a_{1} & a_{4} & a_{6}\\ a_{4} & a_{2} & a_{5}\\ a_{6} & a_{5} & a_{3} \end{array}\right] \end{array}$$

The objective function of Equation (5) is reformulated with the vector a as follows:

(7) $$\begin{array}{l} \underset{j}{\sum }\left\{p_{j}^{T}Ap_{j}-n_{k}^{T}p_{j}\right\}\\ =\underset{j}{\sum }\left\{f\left(p_{j}\right)a-n_{k}^{T}p_{j}\right\}\\ ={\left(F\left(p_{1:N}\right)a-Pn_{k}\right)}^{T}\left(F\left(p_{1:N}\right)a-Pn_{k}\right) \end{array}$$

where:

$$f\left(p\right)≜\left[\begin{array}{cccccc} x^{2} & y^{2} & z^{2} & 2xy & 2yz & 2zx \end{array}\right]\;F\left(p_{1:N}\right)≜\left[\begin{array}{c} f\left(p_{1}\right)\\ f\left(p_{2}\right)\\ \vdots \\ f\left(p_{N}\right) \end{array}\right]\;and\;P≜{\left[\begin{array}{cccc} p_{1}^{T} & p_{2}^{T} & \ldots & p_{N}^{T} \end{array}\right]}^{T}.$$

Similarly, the constraint equation is rewritten as $An_{k}=G\left(n_{k}\right)a=0_{3\times 1}$ where:

$$G\left(n_{k}\right)≜\left[\begin{array}{cccccc} n_{k,x} & 0 & 0 & n_{k,y} & 0 & n_{k,z}\\ 0 & n_{k,y} & 0 & n_{k,x} & n_{k,z} & 0\\ 0 & 0 & n_{k,z} & 0 & n_{k,y} & n_{k,x} \end{array}\right].$$

The final optimization formula is rearranged as the following equation:

(8) $$\begin{array}{l} a=\underset{a}{\arg min}{\left(F\left(p_{1:N}\right)a-Pn_{k}\right)}^{T}\left(F\left(p_{1:N}\right)a-Pn_{k}\right)\\ subject\;to\;G\left(n_{k}\right)a=0_{3\times 1} \end{array}$$

The QP problem in this form is solved by the closed-form formula:

(9) $$\begin{array}{l} \left[\begin{array}{cc} F{\left(p_{1:N}\right)}^{T}F\left(p_{1:N}\right) & G{\left(n_{k}\right)}^{T}\\ G\left(n_{k}\right) & 0_{3\times 3} \end{array}\right]\left[\begin{array}{c} a\\ \lambda \end{array}\right]\\ =\left[\begin{array}{c} F{\left(p_{1:N}\right)}^{T}Pn_{k}\\ 0_{3\times 1} \end{array}\right]. \end{array}$$

The matrix A is reconstructed from the solution a as in Equation (6) and decomposed to obtain $C_{\alpha }$, $C_{\beta }$, and R, which are the principal curvatures at $p_{k}$, and the rotation matrix of which columns are principal axes, respectively.

### 3.2. Curvatures with Gradients

Since real-world surfaces are not always symmetric like quadratic surfaces, two curvature values are not sufficient to describe skewed shapes. To cover the remaining degree of freedom of local surfaces, we estimated the gradients of curvatures. The gradients were estimated by linear regression of the curvatures along the two principal axes, $v_{1}$ and $v_{2}$. The primary curvature around the keypoint can be modeled as:

(10) $$C_{\alpha }=D_{\alpha }v_{1}\cdot \left(p_{1}-p_{k}\right)+c+\varepsilon$$

where $D_{\alpha }$, c, and ε are the gradients of the primary curvature, offset, and fitting error, respectively. The gradient that minimizes the sum of the fitting errors is computed by solving:

(11) $$\left[\begin{array}{cc} v_{1}\cdot \left(p_{1}-p_{k}\right) & 1\\ v_{1}\cdot \left(p_{2}-p_{k}\right) & 1\\ \vdots & \vdots \\ v_{1}\cdot \left(p_{N}-p_{k}\right) & 1 \end{array}\right]\left[\begin{array}{c} D_{\alpha ,k}\\ c \end{array}\right]=\left[\begin{array}{c} C_{\alpha ,1}\\ C_{\alpha ,2}\\ \vdots \\ C_{\alpha ,N} \end{array}\right]$$

where $C_{\alpha ,j}$ is the primary curvature at the *j*th neighbor point. Similarly, the gradient of the secondary curvature, $D_{\beta }$, is estimated by the linear regression over $C_{\beta }$ along $v_{2}$. While both curvature values are symmetric over positive or negative directions of principal axes, the signs of gradients differ with the directions of the principal axes. For consistency of gradient values, the direction of $v_{1}$ was always selected to make $D_{\alpha }$ positive. Then $v_{2}$ was determined by $v_{2}=n_{k}\times v_{1}$. Therefore, our descriptor, PCWG, is presented as $d_{k}=\left[\begin{array}{cccc} C_{\alpha } & C_{\beta } & D_{\alpha } & D_{\beta } \end{array}\right]$. This descriptor expresses the sharpness of a shape by curvatures and the skewness by gradients. Generally, there are three types of shapes: (1) high gradient (the point is between flat and curvy regions); (2) low gradient and high curvature (the point is at the peak of the curve); and (3) low gradient and low curvature (the point is on the plane). We prove in Section 3.3 that these four parameters are sufficient to accurately describe local point clouds.

### 3.3. Gradient Weight

**Figure 2:**
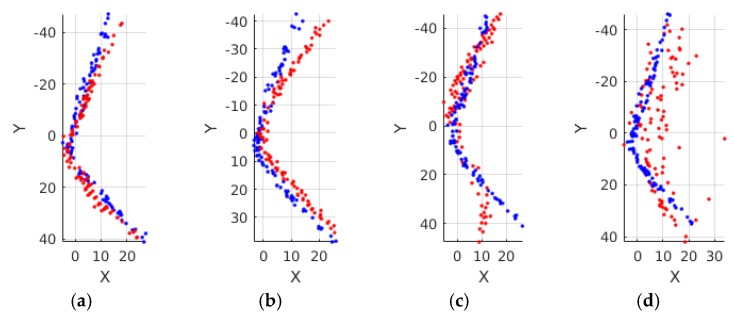
Comparison of different shapes with shape distances. The blue dots represent the reference shape which is the same in the four figures while the red dots represent the different compared shapes. The corresponding shape distances are denoted by $S_{ik}$ below the figures. (**a**) $S_{ik}=1.88$; (**b**) $S_{ik}=3.11$; (**c**) $S_{ik}=5.98$; (**d**) $S_{ik}=9.10$.

Although the gradient terms are auxiliary compared with the curvature terms of our PCWG descriptor, their values readily become larger than the curvatures and are even sensitive to noise, which makes the distance between descriptors to be distorted. As the two terms represent different properties, they do not have to be equally treated. Thus, the gradients should be weighted to suppress their effect on descriptor distances. Ideally, the distance between descriptors should be linearly related to the shape distance. The shape distance is defined as:

(12) $$S_{ik}=D\left({\mathbb{P}}_{i},{\mathbb{P}}_{k}\right)+\eta A\left({\mathbb{N}}_{i},{\mathbb{N}}_{k}\right)$$

where ${\mathbb{P}}_{i}$ and ${\mathbb{N}}_{i}$ are the point cloud and normal vectors at the frame i, respectively, and $D\left({\mathbb{P}}_{i},{\mathbb{P}}_{k}\right)$ is the mean point-to-plane distance between two point clouds, $A\left({\mathbb{N}}_{i},{\mathbb{N}}_{k}\right)$ is the mean angular difference between the normal vector pairs of the point clouds, and η is the angular difference weight for a balance with the point-to-plane distance, which is set to 0.01. To convince the effectiveness of the distance metric, four different shapes (red) are compared with the reference shape (blue) in Figure 2 with the corresponding shape distances. From left to right, the compared shapes differs more with the reference shape with increasing shape distances. The effect of the angular difference weight on performances of descriptors is addressed in Section 4.5.

Based on the shape distance metric, the gradient weight, w, needs to be adjusted to fit the linear regression $\left(d_{k}-d_{i}\right)\cdot w=S_{ik}$ where $w=\left[\begin{array}{cccc} \nu & \nu & \omega & \omega \end{array}\right]$. The descriptor has different weights for the curvatures and gradients. Given a set of pairs of descriptors or local shapes, the weight can be optimized between the descriptor difference and the corresponding shape distance. The optimization problem is defined as:

(13) $$\begin{array}{l} \left(\nu ,\omega \right)=\underset{\nu ,\omega }{\arg\;\min}\underset{i,k}{\sum }\left\{\left(d_{k}-d_{i}\right)\cdot w/S_{ik}-1\right\}\\ \;subject\;to\;\nu ,\omega >0 \end{array}.$$

The relative weight for the gradient terms is calculated as $\overset{´}{\omega }=\omega /\nu$. As the optimal weight value varies depending on the data from 0.2 to 0.5, we selected a fixed value of 0.3 for all evaluations in the following section. Therefore, the final form of PCWG is:

(14) $$d_{k}=\left[\begin{array}{cccc} C_{\alpha } & C_{\beta } & \overset{´}{\omega }D_{\alpha } & \overset{´}{\omega }D_{\beta } \end{array}\right].$$

In this section we have introduced how to compute the curvatures and their gradients by using the optimization techniques. The terms seem to be complicated but there are only two equations to solve, Equations (9) and (11), which are closed-form linear equations with no iterations. Besides, it is not computationally complex. Complexity of solving Equation (9) is linear with the number of neighbor points. Computing $F{\left(p_{1:N}\right)}^{T}F\left(p_{1:N}\right)$ is complex as $O\left({\mathrm{ND}}^{2}\right)$ where N is the number of neighbor points and D is the dimensionality of a point, which is a constant 3, and then subsequent solving linear equation (9 dimensional) and eigen decomposition of A (3 × 3 matrix) are finished in a constant time. The processing times with various parameters were measured and discussed in Section 4.2.

## 4. Evaluation Results

To prove the discriminative power of the proposed descriptor, it was compared with the five aforementioned descriptors using the public datasets. The performance of the descriptors was evaluated by multi-level recognition tests. The first level test was on shape recognition. It was a primitive performance for local shape descriptors to see how effectively descriptors distinguish between different local shapes. Thus, it is critical for point cloud registration or point-level association. The second and third level tests were on object instance and category recognitions, respectively, and they measured the statistical robustness of object-level description. Also, invariance of descriptors to noise and scale changes was addressed. We selected three values of 4, 5, and 6 cm for the support radius. A radius less than 4 cm results in unstable normal vectors. On the other hand, a radius larger than 6 cm is also undesirable because it becomes vulnerable to occlusions and cannot describe small shapes. In the following sections, we introduced the public datasets and existing descriptors, and compared them to our descriptor based on the evaluation results.

### 4.1. Datasets

We used three public RGB-D datasets, the CoRBS dataset [37] and the RGB-D Scenes dataset [42] for shape recognition and the RGB-D Object dataset [42] for object recognition. The properties of the datasets for shape recognition were summarized in Table 2. The CoRBS dataset was captured by Kinect v2 and we selected four video sequences which captured four different objects. In Table 2, the numbers after the scene name indicate the video ID to identify a specific video among the videos from the same scenes and the length in the fourth column means the length of a camera trajectory. The RGB-D Scenes dataset was captured by Kinect v1 in home and office environments. We selected five video sequences, which are the first videos from each scene.

**Figure 3:**
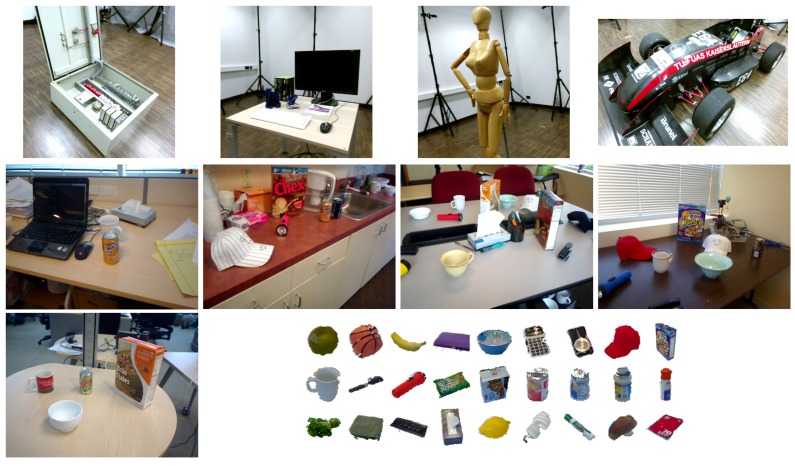
Sample images from the datasets: the top row shows the four objects from the CoRBS dataset; the next five scenes are from the RGB-D Scenes dataset; and the last figure shows exemplar images of RGB-D Object dataset.

The RGB-D Object dataset has a hierarchical structure of video sequences in four levels: category, instance, video, and frames. The dataset contains 300 objects which belongs to 51 categories, and there are multiple videos with hundreds of frames for each object taken at different viewpoints. The main advantage of the dataset is that various items usually observed in a home environment are included. The sample images from the datasets are shown in Figure 3.

### 4.2. Implementation Details

In the implementation, depth images are scaled down to a size of 320 × 240 in order to speed up the processing and suppress noise. As all the datasets provide 640 × 480 images, 2 × 2 depth pixels are averaged as a single depth value. Neighbor points around a keypoint were evenly sampled within the support radius to prevent curvatures from being biased to a denser region within the radius. The number of sampled points was less than or equal to 50. For efficiency of computation, neighbor search results were shared with both normal and descriptor estimation. The three components, neighbor searching, normal estimation, and descriptor estimation were implemented in OpenCL [43]. As the processing time might vary with a support radius and the maximum number of sampled points, we measured the processing time with different combinations of parameters with the GPU device of GTX 1080. The processing times for neighbor searching and normal estimation were less than 1 ms and they are ignorable for any combination of parameters. As normal vectors were commonly used by all the descriptors, we would analyze only the processing time of our descriptor. 

As summarized in Table 3, descriptor estimation took several milliseconds. As expected, the processing time apparently increases with the maximum number of sampled points. The support radius was inversely related to the processing time. That is because the search area is more likely to be occluded with a larger radius and hence the effective number of sampled points decreases. The table shows that the proposed method is fast enough to run in real time regardless of the parameters, even with less powerful devices.

**Figure 4:**
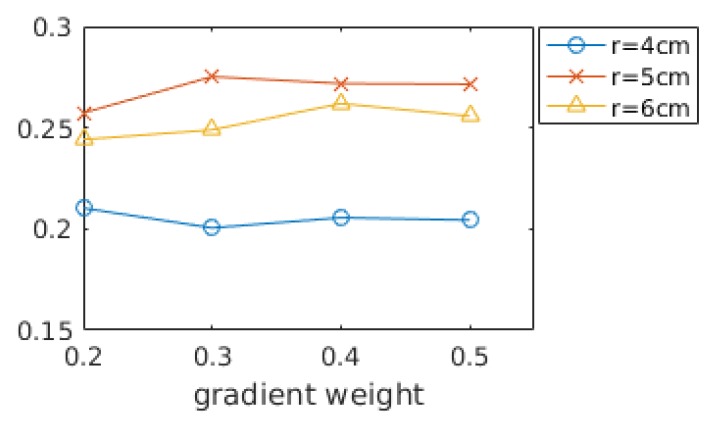
Shape recognition rates with different gradient weights and support radii.

In Section 3.3, the gradient weight $\overset{´}{\omega }$ was optimized by Equation (13) under the assumption that a distance between shape descriptors should be proportional to the corresponding shape distance. To see the validity of the assumption, shape recognition rates are evaluated while varying gradient weights as shown in Figure 4. The shape recognition rate (Precision-1) is defined in Section 4.4 in detail. The shape recognition rates are averaged over all the datasets in Table 2. The performance did not vary much with gradient weight but does with a support radius. As we expected in Section 3.3, the best result is obtained when the gradient weight is 0.3 and the radius is 5 cm. In addition, we can observe that the best radius is 5 cm and the smaller radius (4 cm) is less dependent on the gradient terms than the best radius.

### 4.3. Existing Descriptors

For comparison with the existing descriptors, we used the PCL implementation of FPFH [44], SHOT [16], and Spin Image [14]. Their default dimensionalities are 33, 352, and 153, respectively. TriSI [17] was implemented by computing three spin images along the three principal axes, $v_{1}$, $v_{2}$, and $v_{3}$, and concatenating them as a single vector. Guo et al. reduced the dimensionality of TriSI with the PCA approach, but we did not because the quality of the compression depended on the pre-training data which was difficult to standardize. We used the raw TriSI, and hence its dimensionality became 459. We implemented BRAND [28] by ourselves. The local binary pattern with a radius of 24 pixels was created by Gaussian distribution of $N\left(0,48^{2}/25\right)$ and texture information was not used but only geometric information was used for binary tests. The PCWG descriptor was also compared with the pure principal curvatures (PC) with a dimensionality of 2. Therefore, seven types of descriptors were compared in total.

### 4.4. Shape Recognition

As mentioned in Section 2.4, keypoint matching does not work effectively because local shapes are ambiguous. Instead, we developed a new method to evaluate local shape recognition performance. To evaluate how well the descriptors recognize a similar shape among various shapes, we needed to extract a set of *representative* shapes which are both frequently observed and as diverse as possible. 

We extracted two sets of representative descriptors from each dataset: one was a reference set, and the other was a query set. The sets of pairs of representative descriptors and shapes were used to evaluate shape recognition rates. The samples of representative shapes were shown in Figure 2 where the blue shape is the reference shape and the red shapes are from the query set.

The representative shapes were found by clustering a huge pool of shape descriptors and selecting the centroids of the clusters. For fair competition among the descriptors, a concatenation of PCWG, FPFH, SHOT, and TriSI descriptors, named as a total descriptor, was used for the clustering. The principal curvatures and Spin Image were not used because they are included in the PCWG and TriSI descriptors, respectively. As naive clustering of the total descriptors is prone to be biased to dominant shapes (flat shapes), the iterative clustering with following steps was used:

1. Total descriptors are computed at the sampled points in frames of a video sequence.
2. Total descriptors are grouped by K-means clustering.
3. The dominant clusters are resampled to reduce the population.
4. Iterate from 2, until no dominant cluster exists.

In the first step, tens of points with more than 25 neighbor points were evenly sampled except for large planar regions in each frame. The number of clusters, K, was 100 in the second step. The dominant cluster was defined as a cluster with population more than 4T/K, where T is the number of the total descriptors. In the third step, the dominant clusters were reduced to 2T/M. To obtain the two sets of descriptors, the reference set was extracted first, and then the query set was selected after excluding the reference set from the pool of descriptors.

Given the two sets of representative descriptors, the correspondence to a queried descriptor was predicted by finding the closest descriptor in the reference set. Precision-1 refers to the ratio of the correspondences where the closest descriptor was from the closest shape in terms of the shape distance of Equation (12). Precision-5 means the ratio of the correspondences where the closest descriptor belongs to the five closest shapes. Correspondences with shape distances larger than the support radius were rejected in the evaluation. The two metrics were computed for the seven descriptors for the same sets of representative shapes.

**Figure 5:**
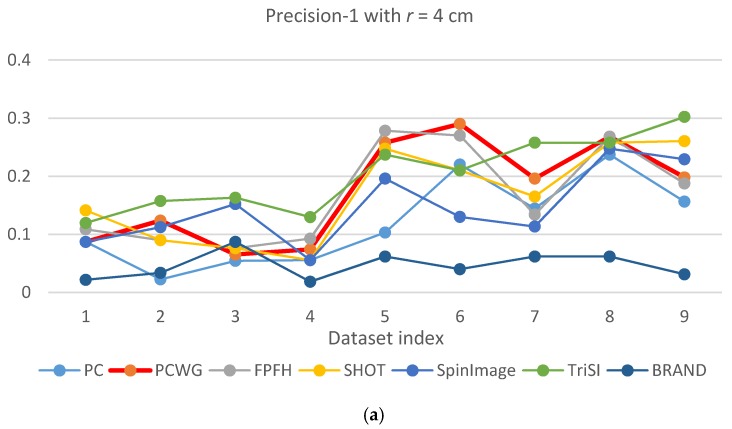

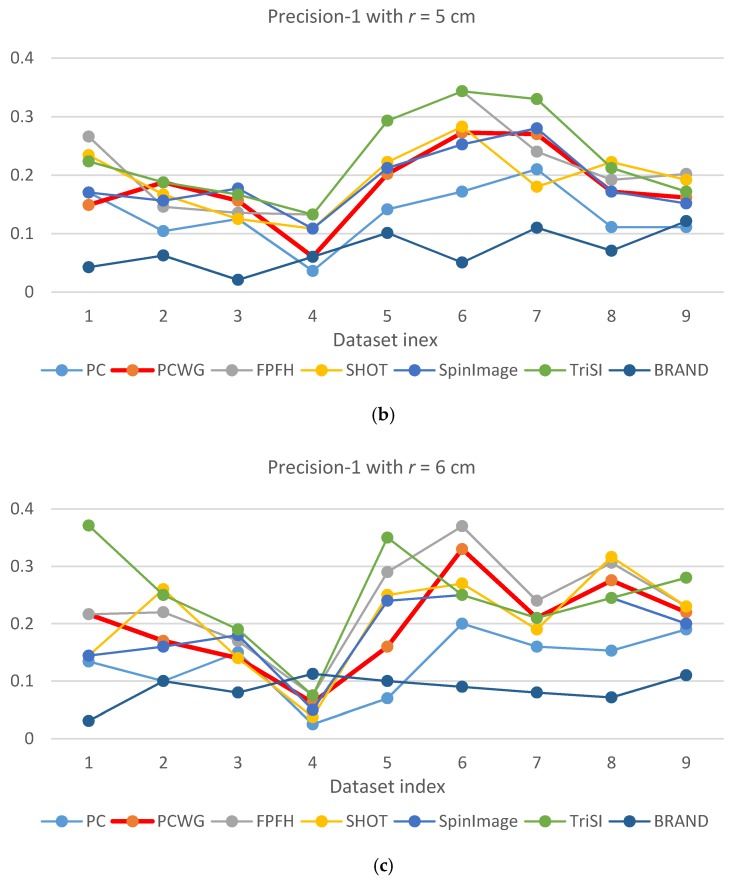
Precision-1 comparison of seven descriptors over nine datasets with different support radii: (**a**) *r* = 4 cm, (**b**) *r* = 5 cm, and (**c**) *r* = 6 cm.

**Figure 6:**
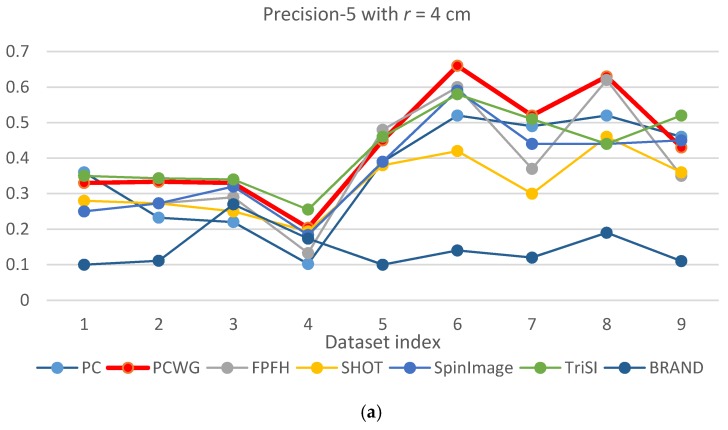

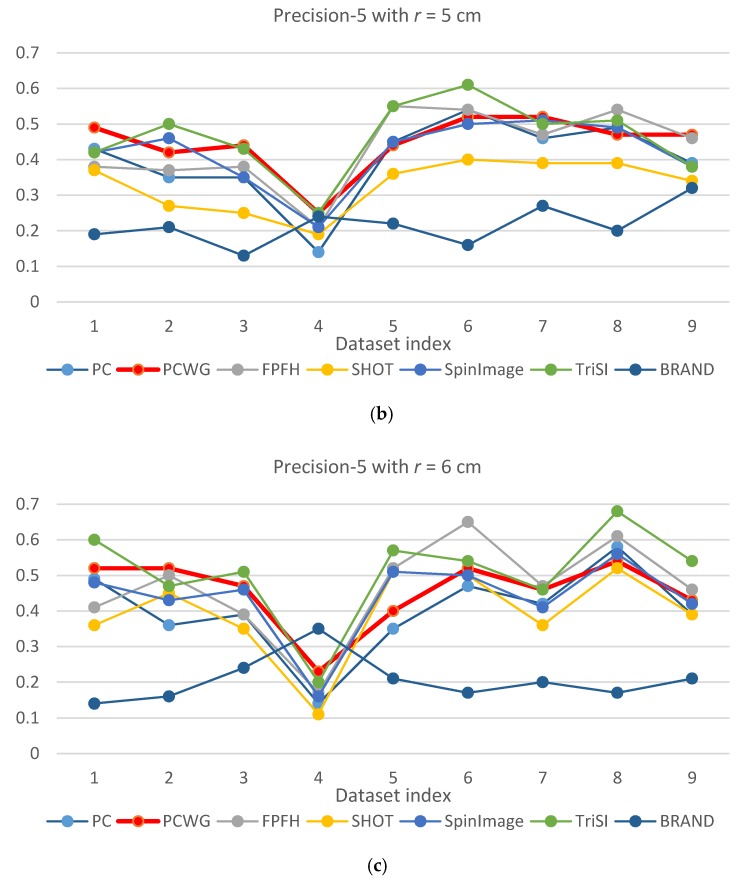
Precision-5 comparison of seven descriptors over nine datasets with different support radii: (**a**) *r* = 4 cm, (**b**) *r* = 5 cm, and (**c**) *r* = 6 cm.

Figure 5 and Figure 6 show the evaluation results of Precision-1 and Precision-5, respectively, over the eight datasets with the three different support radii. In the figures, PCWG was denoted by the bold red lines. Overall, PCWG stayed in the middle of other descriptors. It means that PCWG’s performance is comparable with the others despite its extremely low dimensionality. That is confirmed by Table 4 and Table 5 where precision-1 and -5 were averaged over the nine datasets for each support radius and the last column shows the ranking of PCWG. In the tables, it is noteworthy that the relative performance of PCWG with a radius of 4 cm was ranked second and first in Precision-1 and Precision-5, respectively. It indicates that PCWG is good at roughly searching small shapes.

The best overall performance came from TriSI which is the most high-dimensional descriptor but the second best was FPFH of which dimensionality is just 33. On the other hand, the performance of SHOT and BRAND were disappointing since SHOT has the second largest dimensionality, 352, and BRAND was reported that it performed better than CSHOT or Spin Image in [28]. The reason seems to be that texture information was not adopted into BRAND in our implementation. For BRAND, there were so many equally distanced shapes because it used the hamming distance while the others use a floating-point L1 distance.

There is another notable point that precisions of most descriptors were generally low in the racing car dataset, especially when a support radius is small. The reason seems to be the scale of the object (racing car). In Figure 3, the racing car looks like it contains various shapes but the scale of the shapes are larger than the support radii. Since local shapes within the radii, 4 to 6 cm, were not distinctive enough in the dataset, the shape recognition rates generally fell down except for BRAND.

From the shape recognition results, we can conclude that the performance of descriptors does not depend on dimensionality, and our compact descriptor can work as effectively as high dimensional descriptors when querying shape from depth images.

### 4.5. Effect of Angular Difference Weight

**Figure 7:**
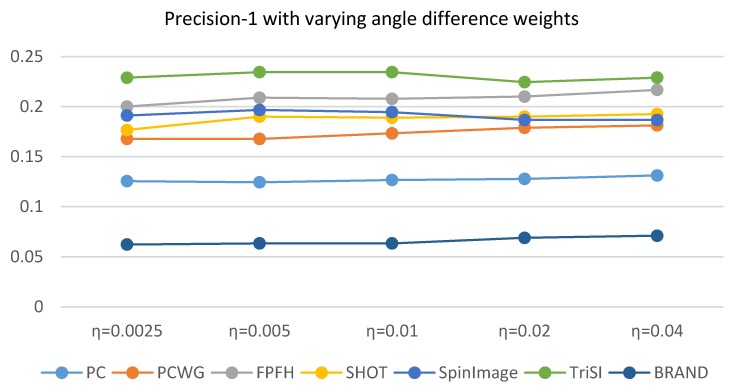
Comparison of Precision-1 of the seven descriptors where the precision was averaged over the nine datasets.

The effect of the angular difference weight, η, in Equation (12) is addressed here. This parameter balances the point-to-plane distance and the angular difference. Since the shape distance was used to find ground truth correspondences for shape recognition, the parameter should be carefully selected but it is more desirable that the shape recognition rate is insensitive to the parameter. We evaluated Precision-1 with different values of the parameter. For simplicity, a single support radius, 5 cm, was used and the precisions over the nine datasets were averaged. The results were shown in Figure 7. While the angular difference weight varied from 0.0025 to 0.04, the precisions differed just 1 or 2 percentages. As the shape recognition rate is not sensitive to the angular difference weight, our shape distance metric with η=0.01 can be considered to be generally reliable to find ground truth correspondences and not biased to any descriptor.

### 4.6. Robustness to Noise and Scale Variation

**Figure 8:**
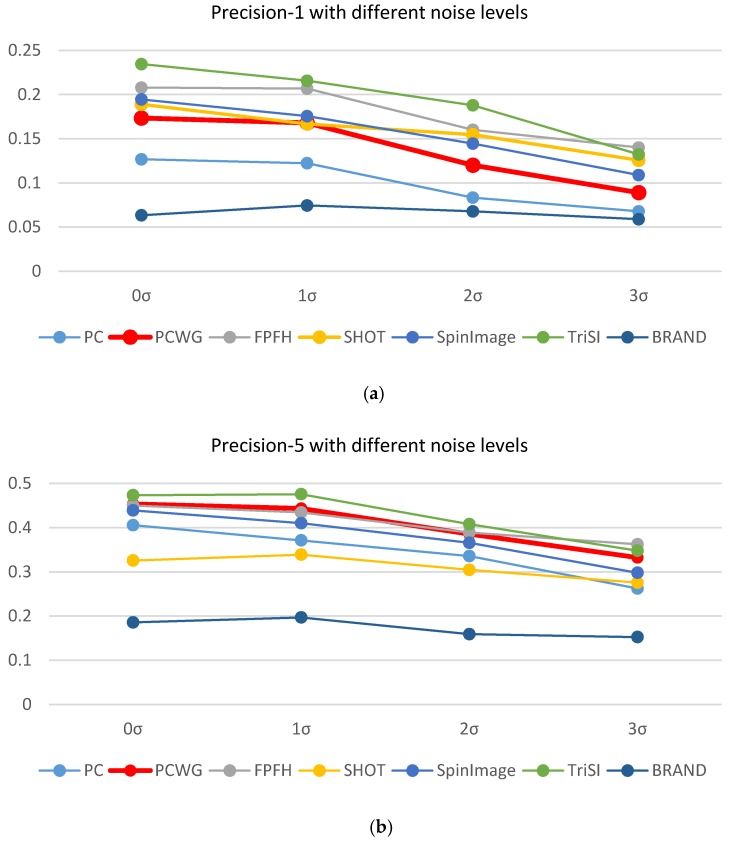
Precisions of seven descriptors with different noise levels. The horizontal axis represents the standard deviation of additive noise to raw depth images. 0 σ means that raw depth images were used. (**a**) Precision-1 and (**b**) Precision-5.

An ideal descriptor should be invariant to noise and scale changes. In order to evaluate the robustness of descriptors, shape recognition rates were recomputed with additive noise or scaled depth images. To simplify the test, we used a single support radius of 5 cm. Robustness to noise was evaluated by adding Gaussian noise to depth images. Wasenmüller et al. [45] showed the graph where the standard deviation of noise of Kinect v2 increased with a depth, which could be approximately modeled by the following linear equation:

(15) σ(d)=1.3*d+0.3

where σ(d) represents the standard deviation of noise in millimeters at a depth, d. To simulate amplifying noise, we added noise with the standard deviation of τσ(d) to raw depth images. In our simulation, shape recognition rates were re-evaluated with different noise levels of τ=0, 1, 2, 3 and the results were shown in Figure 8 where precisions were averaged over the nine datasets. As expected, the both precisions tended to decrease with increasing noise levels regardless of descriptor types. The slopes of decrements did not differ much among the top-5 descriptors in the both precisions. Though curvatures and even its gradients of surfaces are known to be sensitive to noise, the graph of PCWG is blended with other descriptors in the figure. Thus the PCWG’s robustness to noise is similar to the existing descriptors.

Another issue is scale invariance. Image scale of an object changes with a distance. Ideally, shape descriptors should not affected by distance when they describe shapes within the same physical radius at the same location. However, descriptors can be influenced by scale changes in reality because both image resolution and noise property vary with a distance. As a camera position changes, we cannot re-compute a descriptor at the exactly the same position with the previous position, where a descriptor was computed, but only at the closest position in the current point cloud. As this slight error as well as sensor random noise affects the normal vector at the point, descriptor may change.

To simulate robustness of descriptors to scale changes, we computed descriptors at different image scales. As commented in Section 4.2, we used scaled images of 320 × 240 resolution in our experiment where the raw images were at 640 × 480 resolution. The reference descriptor set in Section 4.4 was re-computed at both double-scale (640 × 480) and half-scale (160 × 120) images. These *scaled* descriptors were used as the query descriptor sets and the shape recognition rates were evaluated as summarized in Table 6. The precisions were averaged over the nine datasets. FPFH and SHOT showed the best results on average while PCWG was ranked low. Overall, descriptors tended to be more robust when a scale was reduced except for FPFH. Similar to the results in the previous section, BRAND showed the lowest performance. We counted binary ‘one’s in BRAND at different scales, and BRAND contained about 25 ones at mid and high resolution and 10 ones at low resolution on average. The number of ones was largely affected by image resolution, which explains the result. It seems that PCWG was less robust to scale changes because the curvatures are highly sensitive to variation in normal vectors. The other descriptors are also largely dependent on normal vectors, but the effect of a normal vector is weakened by quantization of property values and smoothing histograms in descriptors. On the other hand, curvatures are directly influenced by normal vectors. However, gradient terms in PCWG helped the robustness, compared to PC and PCWG showed almost comparable results in Precision-5.

### 4.7. Object Recognition

Object recognition is another important application of local shape descriptors. The six types of descriptors out of the seven were compared in object recognition except for BRAND, which showed meaninglessly low performance in the shape recognition. For object recognition, a typical bag-of-words (BoW) approach [46] was used. As our aim was not to achieve a higher recognition rate but to compare relative performances of descriptors, we used neither an SVM classifier nor the weighted distance [47] but used simple L1 distance for recognition. More advanced BoW techniques may result in higher recognition rates but if the performances of all the descriptors are improved, relative results will be the same. In addition, this simple classifier could not be optimized for any descriptor and is easy to implement and fast. To train code words, descriptors from the very first videos of all instances in the RGB-D object dataset were clustered for each type of descriptor. Objects were recognized in two levels, instance and category. In each level, recognition rates were evaluated with all the combinations of three codebook sizes (50, 100 and 200) and the support radii.

For object instance recognition, the video-level BoW descriptor was computed by averaging the BoW descriptors of the first five frames of the video. Two sets of video-level BoW descriptors were constructed. As multiple videos belong to each instance, one set is from the second videos of all instances and the other set is from the third videos. The performance of instance recognition was evaluated by cross validation between the two sets of video-level BoW descriptors.

For category recognition, the instance-level BoW descriptor was obtained by averaging the video-level descriptors belonging to the same instance. For each category, the instance-level descriptors were computed from five instances belonging to the category. Three of them were selected to model the reference category-level BoW descriptor by averaging the selected instance-level descriptors. The other two instance-level descriptors were matched with the closest category-level descriptor for category recognition. For cross validation, the initial selection of instances for the category-level descriptor was the three consecutive instances beginning from the first one, and the selection kept to be shifted to begin with the next one. Total ten tests were made for each category from two query instances for each of the five selections.

**Figure 9:**
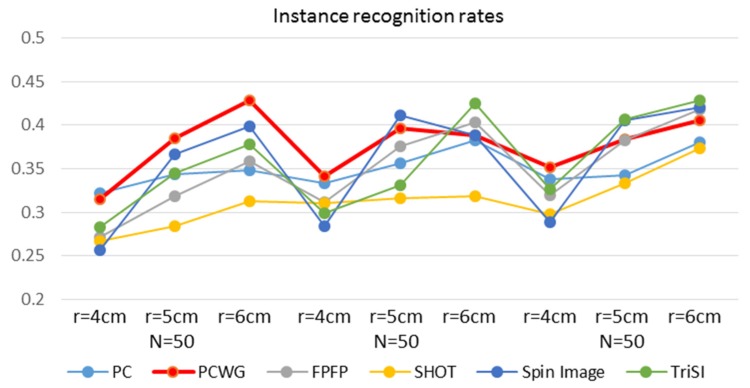
Instance recognition rates of six descriptors with different codebook sizes and support radii.

**Figure 10:**
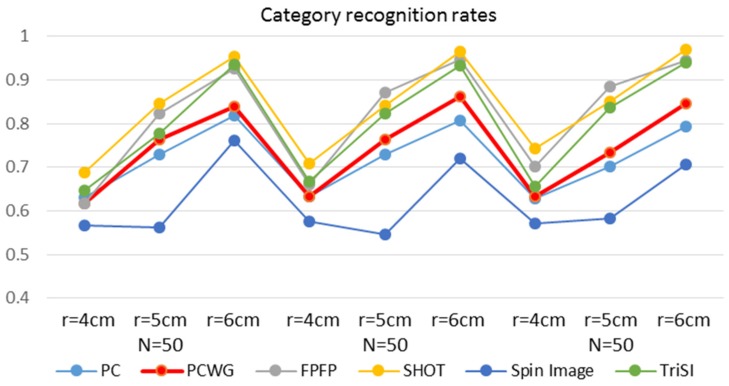
Category recognition rates of six descriptors with different codebook sizes and support radii.

Figure 9 and Figure 10 show the instance and category recognition results, respectively. Generally, PCWG ranked high in instance recognition but low in category recognition, and surprisingly, the simplest PC also showed the meaningful performance in instance recognition. On the contrary, SHOT showed the best performance in the category recognition but the worst in instance recognition. It seems that PCWG was better in matching specific shapes, while SHOT was better in the generalization of shapes. Spin Image showed generally low performance in category recognition. It is noteworthy that the both performances tend to increase with the radius in most of the descriptors. It is more apparent in the category recognition.

Overall, the superiority of PCWG in instance recognition points out the large redundancy of the existing descriptors, while the inferiority in category recognition reveals an excessive sensitivity to small changes in shapes.

## 5. Conclusions

Since local shape descriptors have lower performances than local texture descriptors in general, their large dimensionalities motivated us to figure out the origin of high dimensionalities and an alternative compact descriptor with comparable performance. The answer to the question in the title is here: High dimensional descriptors have a potential to discriminate various shapes, especially when the shape is complicated and densely modeled. However, they usually waste memory dealing with depth images from Kinect-like popular sensors where the shapes are uncomplicated and the sensor resolutions are limited.

That is why we proposed a new descriptor, PCWG. The principal curvatures roughly describe a shape as a quadratic surface and their gradients add the details of the shape. Closed-form equations were derived to estimate the descriptor from the point cloud, and we implemented it based on GPU. We proved the inefficiency of the existing descriptors in Section 2 and showed the competitive performance of the PCWG in Section 4. Although the proposed descriptor is only four dimensional, it showed superior performance in shape and object instance recognition with a small support radius, a medium performance with larger support radii, and lower performance in object category recognition. The PCWG’s high sensitivity to shapes is advantageous for low-level shape matching but disadvantageous for shape abstraction. This is because a small change in a part of a local shape affects the entire vector of the PCWG, while it affects only a part of the histogram-based descriptors. 

In conclusion, our descriptor is useful for point association and object instance recognition in ordinary environments with limited resources. For the future works, we will develop a more advanced descriptor to express more complicated shapes with the least additional dimensions. For instance, texture information can be adopted to make up for the simplicity of PCWG. The extension of PCWG could lead to a new descriptor, which satisfies both the better performance in all recognition levels and the new approaches in Section 2.4.

## Figures and Tables

**Table 1:** Number of effective principal components.

		FPFH	SHOT	Spin Image	TriSI	BRAND
Dimensionality		33	352	153	459	256
Cabinet	nEPC	7	60	30	54	205
	rEPC (%)	21.2	17.0	19.6	11.8	44.7
Desktop	nEPC	7	59	34	60	205
	rEPC (%)	21.2	16.8	22.2	13.1	44.7
Human	nEPC	8	56	35	64	205
	rEPC (%)	24.2	15.9	22.9	13.9	44.7

**Table 2:** The properties of the datasets used for shape recognition.

Dataset (Sensor)	Scene Name	Dataset Index	Length (m)	# Frames
CoRBS (Kinect v2)	Electrical cabinet #2	1	23.0	1902
	Desk #2	2	11.5	2380
	Human #2	3	11.3	2547
	Racing car #2	4	34.1	3209
RGB-D Scenes (Kinect v1)	Desk #1	5	N/A	98
	Kitchen_small #1	6	N/A	180
	Meeting_small #1	7	N/A	180
	Table #1	8	N/A	125
	Table_small #1	9	N/A	199

**Table 3:** Processing time (ms) of PCWG estimation vs. the support radius and the maximum number of sampled points.

Radius\#points	30	40	50
4	3.53	4.13	4.64
5	3.21	3.79	4.06
6	3.18	3.75	4.03

**Table 4:** Average precision-1 for three support radii.

Radius (cm)	PC	PCWG	FPFH	SHOT	SpinImage	TriSI	BRAND	Rank
4	0.120	0.173	0.167	0.167	0.147	0.204	0.046	2
5	0.131	0.181	0.217	0.193	0.187	0.229	0.071	5
6	0.131	0.198	0.235	0.204	0.187	0.247	0.086	4

**Table 5:** Average precision-5 for three support radii.

Radius (cm)	PC	PCWG	FPFH	SHOT	SpinImage	TriSI	BRAND	Rank
4	0.366	0.432	0.374	0.324	0.371	0.422	0.146	1
5	0.400	0.447	0.433	0.329	0.419	0.461	0.216	2
6	0.399	0.454	0.464	0.394	0.437	0.508	0.206	3

**Table 6:** Shape recognition rates between different image scales.

Scale	Precision	PC	PCWG	FPFH	SHOT	SpinImage	TriSI	BRAND
1/2	Precision-1	0.538	0.622	0.668	0.917	0.946	0.860	0.025
1/2	Precision-5	0.888	0.871	0.835	0.950	0.985	0.939	0.090
2	Precision-1	0.246	0.318	0.775	0.569	0.341	0.426	0.020
2	Precision-5	0.602	0.645	0.947	0.721	0.664	0.711	0.094
